# Editorial: Functional Testing of Vestibular Function

**DOI:** 10.3389/fneur.2020.00654

**Published:** 2020-06-30

**Authors:** Michael C. Schubert, Stefano Ramat, Herman Kingma

**Affiliations:** ^1^Laboratory of Vestibular NeuroAdaptation, Otolaryngology Head and Neck Surgery, Johns Hopkins University, Baltimore, MD, United States; ^2^Department of Electrical, Computer and Biomedical Engineering, University of Pavia, Pavia, Italy; ^3^Department of Otolaryngology, Maastricht, Netherlands; ^4^Department of Medical Physics, Tomsk Research State University, Tomsk, Russia

**Keywords:** vestibular function, vestibulo-ocluar reflex, gaze stability, balance, head impulse

The vestibular ocular reflex is responsible for stabilizing the eyes in space (known as gaze stability) and the body in space (postural and gait stability) during head motion. This is achieved initially by generating exceptionally short latency vestibular oculomotor reflexes (~10 ms) of the same magnitude and velocity, but in the opposite direction of the sensed head motion. The head motion signal also descends via the vestibulo-spinal system for postural and gait stability. Knowledge of this reflexive function has spawned the development of tests and measures that assay the behavioral relevance of vestibular sensation, without measuring the physiologic performance (i.e., measure gaze stability without recording the movement of the eyes). The focus of this special topic is on the diagnostic accuracy and rehabilitative efforts using novel tests and measures of vestibular behavior.

Millar et al. illustrate that vestibular rehabilitation improves gaze stability and other functional outcome measures, but that such recovery correlates with residual otolith function not change in semicircular canal function. Similarly, Allum et al. report that balance rehabilitation can restore normal balance 3 months after acute bilateral semicircular canal loss as long as otolith function is spared.

Using the novel functional head impulse test (fHIT), Romano et al. report that athletes of different sports have different gaze stabilization performances when tested at the highest head angular accelerations. van Dooren et al. compared the fHIT with dynamic visual acuity during gait on a treadmill and quantify the oscillopsia in patients with bilateral vestibular hypofunction to report that the fHIT correlates with experienced oscillopsia as measured by the Oscillopsia Severity Questionnaire. Sjögren et al. investigated nine patients with total unilateral vestibular loss using passive and active head impulses and the fHIT. They report normal VOR performance toward the affected side during active head impulses and that visual acuity as measured by the fHIT correlated with the latency of covert saccades.

Ramaioli et al. compared dynamic visual acuity during translational and angular head motion in a group of normal subjects and report that the well-known under-compensatory translational VOR gains are correlated with worse DVA scores during translational head motion.

Figtree and Migliaccio propose an interesting low-cost motion tracking system for posturography based on a stereo vision system that tracks a body-fixed pattern of markers. Coupled to a force plate it represents an affordable alternative for functional assessment of balance abilities.

Dasgupta and Ratnayake conducted a retrospective review of superior canal dehiscence (SCD) in children and correlated functional (audiometry), behavioral (eye movements), and anatomical (computed tomography scan) findings to reveal that children with SCD show phenotypes different from those reported in adults.

Fu et al. studied the high acceleration VOR in a set of 47 patients with acute vestibular neuritis using the video head impulse test and the dizziness handicap inventory (DHI). At 6 months follow-up, the patients had significant reduction in compensatory saccades (overt and covert), but also that the patients with normal to mild DHI scores (DHI ≤ 30) had higher VOR gain and lower number of covert saccades.

Taken together, this collection of papers underlines the importance of functional testing for quantifying the ecological ability of patients with vestibular hypofunction, and reveals the importance of how errant vestibular information impacts quality of life. Functional testing appears especially useful for assessing the effectiveness of rehabilitation programs, where the functional ability of the patient is more concerning and may be more informative than the objective measurement *per se* (e.g., VOR gain). The plasticity of our nervous system indeed is challenged by vestibular failure. The brain expends a fascinating repertoire of mechanisms to ensure functional navigation of everyday life, be it via recalibration or substitution.

## Author Contributions

All authors listed have made a substantial, direct and intellectual contribution to the work, and approved it for publication.

## Conflict of Interest

The authors declare that the research was conducted in the absence of any commercial or financial relationships that could be construed as a potential conflict of interest.

